# Supplementary material for: Terpenoids from the Seeds of Toona sinensis and Their Ability to Attenuate High Glucose-Induced Oxidative Stress and Inflammation in Rat Glomerular Mesangial Cells
Source: Molecules. 2022 Sep 7;27(18):5784. doi: 10.3390/molecules27185784 (PMC9503114; doi:10.3390/molecules27185784)
Supplement: Supplementary file 1 [file molecules-27-05784-s001.zip › molecules-1859556-supplementary.pdf]

Supplementary Materials

Terpenoids from the seeds of *Toona sinensis* and their ability to attenuate high glucose-induced oxidative stress and inflammation in rat glomerular mesangial cells

Ying Chen †, Hong Gao †, Xiaoxiao Liu †, Jinyi Zhou, Yijin Jiang, Feng Wang, Rongshen Wang *, Wanzhong Li *

School of Pharmacy, Weifang Medical University, Weifang 261053, P. R. China

Abstract: *Toona sinensis* (A. Juss.) Roem is an edible medicinal plant that belongs to the genus *Toona* in the Meliaceae family. It has been confirmed to display a wide variety of biological activities. During our continuous search for active constituents from the seeds of *T. sinensis*, 2 new acyclic diterpenoids (**1**–**2**), together with 5 known limonoid-type triterpenoids (**3**–**7**), 5 known apotirucallane-type triterpenoids (**8**–**12**), and 3 known cycloartane-type triterpenoids (**13**–**15**), were isolated and characterized. Their structures were identified based on extensive spectroscopic experiments including nuclear magnetic resonance (NMR), high-resolution electrospray ionisation mass spectra (HR-ESI-MS), and electronic circular dichroism (ECD), as well as the comparison with those reported in the literature. We compared these findings to those reported in the literature. Compounds **5**, **8**, and **13**–**14** were isolated from the genus *Toona*, and compounds **11** and **15** were obtained from *T. sinensis* for the first time. The antidiabetic nephropathy effects of isolated compounds against high glucose-induced oxidative stress and inflammation in rat glomerular mesangial cells (GMCs) were assessed *in vitro*. The results showed that new compounds **1** and **2** could significantly increase the levels of Nrf-2/HO-1 and reduce the levels of NF-κB, TNF-α, and IL-6 at concentrations of 30 μM. These results suggest that compounds **1** and **2** might prevent the occurrence and development of diabetic nephropathy (DN) and facilitate the research and development of new antioxidant and anti-inflammatory drugs suitable for the prevention and treatment of DN.

Keywords: *Toona sinensis* (A. Juss.) Roem; acyclic diterpenoids; rat glomerular mesangial cells; oxidative stress; inflammation

Computational methods

1. Conformational analysis and structure optimization

The conformers of each configuration that were obtained with conformational analysis were then optimized with the software package Gaussian 09 at the M062X/6-31G(d) level. Room-temperature equilibrium populations were calculated according to Boltzmann distribution law (eq. S**1**). The energies and populations of dominative conformers were obtained.

$$\frac{N_i}{N} = \frac{g_i e^{-\frac{E_i}{k_B T}}}{\sum g_i e^{-\frac{E_i}{k_B T}}} \qquad \textbf{(S1)}$$

where N_i is the number of conformer i with energy E_i and degeneracy g_i at temperature *T*, and k_B is Boltzmann constant.

2. NMR calculation

NMR calculations were carried out by Gaussian 09 following the protocol adapted from Lodewyk *et al.* [1]. The theoretical calculation of NMR was conducted using the Gauge-Including Atomic Orbitals (GIAO) method at mPW1PW91/6-31G(d) by the SMD model. Finally, the calculated NMR chemical shift values were averaged according to Boltzmann distribution for each conformer and fitting to the experimental values by linear regression. DP4+ probability analysis was performed according to the reported methods [2].

References

1. Lodewyk, M.W., Siebert, M.R., Tantillo, D.J. Computational prediction of ^{1}H and ^{13}C chemical shifts: a useful tool for natural product, mechanistic, and synthetic organic chemistry. *Chem. Rev.* **2012**, *112*, 1839–1862.

2. Grimblat, N., Zanardi, M.M., Sarotti, A.M. Beyond DP4: an improved probability for the stereochemical assignment of isomeric compounds using quantum chemical calculations of NMR shifts. *J. Org. Chem.* **2015**, *80*, 12526–12534.

List of Figures

Figure S1. The effects of compounds **1−15** (80 μM) on cells viability of GMCs were tested by MTT assay. Values are expressed as mean ± SD of three independent experiments, with $^{**}P <$ 0.01 relative to the NG.

Figure S2. ^{1}H NMR spectrum of compound **1** in $CDCl_3$ (600 MHz)

Figure S3. ^{13}C NMR spectrum of compound **1** in $CDCl_3$ (150 MHz)

Figure S4. DEPT 135° spectrum of compound **1** in $CDCl_3$

Figure S5. ^{1}H−^{1}H COSY spectrum of compound **1** in $CDCl_3$

Figure S6. HMQC spectrum of compound **1** in $CDCl_3$

Figure S7. HMBC spectrum of compound **1** in $CDCl_3$

Figure S8. NOESY spectrum of compound **1** in $CDCl_3$

Figure S9. HR-ESI-MS of compound **1**

Figure S10. ^{1}H NMR spectrum of compound **2** in $CDCl_3$ (600 MHz)

Figure S11. ^{13}C NMR spectrum of compound **2** in $CDCl_3$ (150 MHz)

Figure S12. DEPT 135° spectrum of compound **2** in $CDCl_3$

Figure S13. ^{1}H−^{1}H COSY spectrum of compound **2** in $CDCl_3$

Figure S14. HMQC spectrum of compound **2** in $CDCl_3$

Figure S15. HMBC spectrum of compound **2** in $CDCl_3$

Figure S16. NOESY spectrum of compound **2** in $CDCl_3$

Figure S17. HR-ESI-MS of compound **2**

Figure S18. Chemical structure of the calculated configurations.

Table S1. Statistics of ordinary least squares (OLS) linear regression of experimental and computed ^{13}C-NMR chemical shifts.

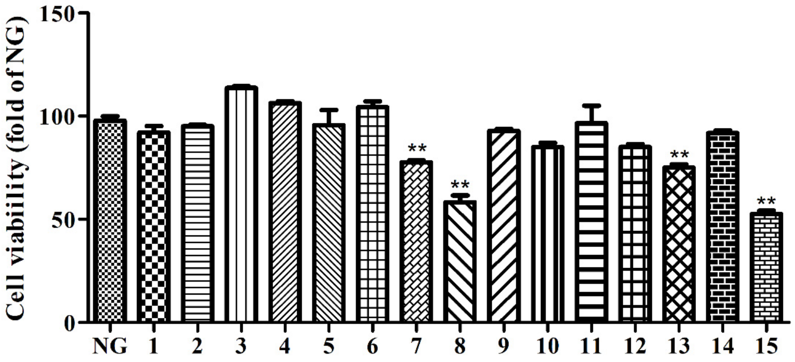

Figure S1. The effects of compounds **1−15** (80 μM) on cells viability of GMCs were tested by MTT assay. Values are expressed as mean ± SD of three independent experiments, with $^{**}P < 0.01$ relative to the NG.

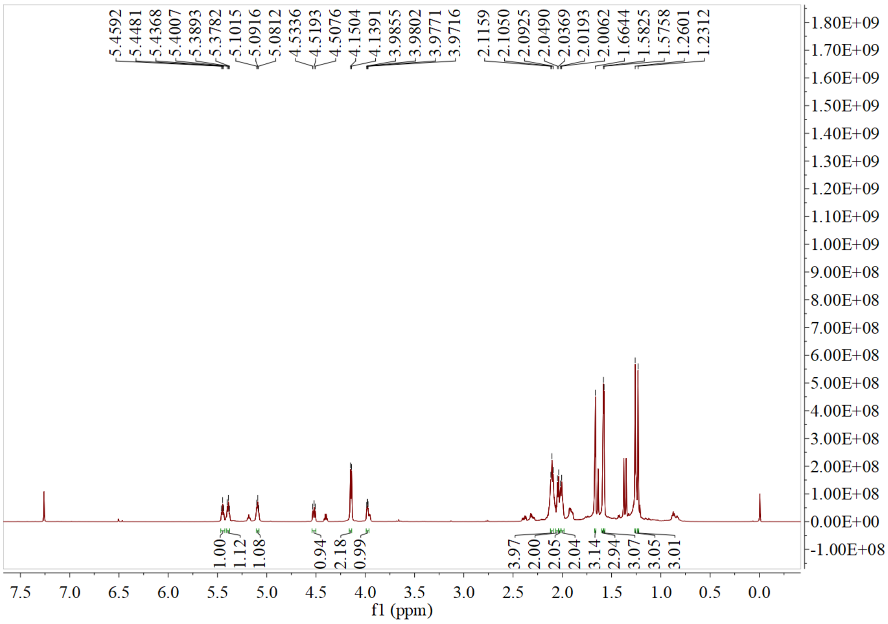

Figure S2. ^{1}H NMR spectrum of compound **1** in $CDCl_3$ (600 MHz)

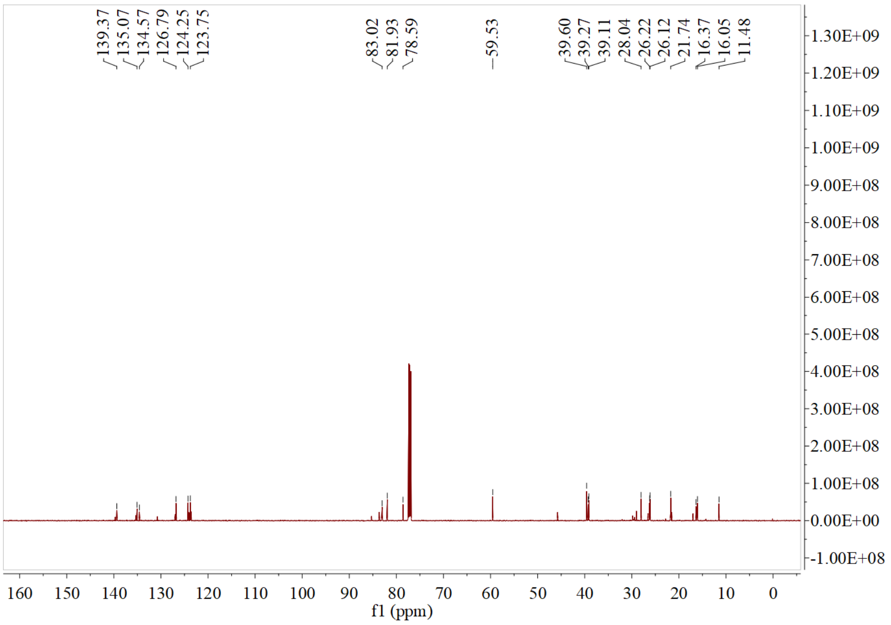

Figure S3. ^{13}C NMR spectrum of compound **1** in $CDCl_3$ (150 MHz)

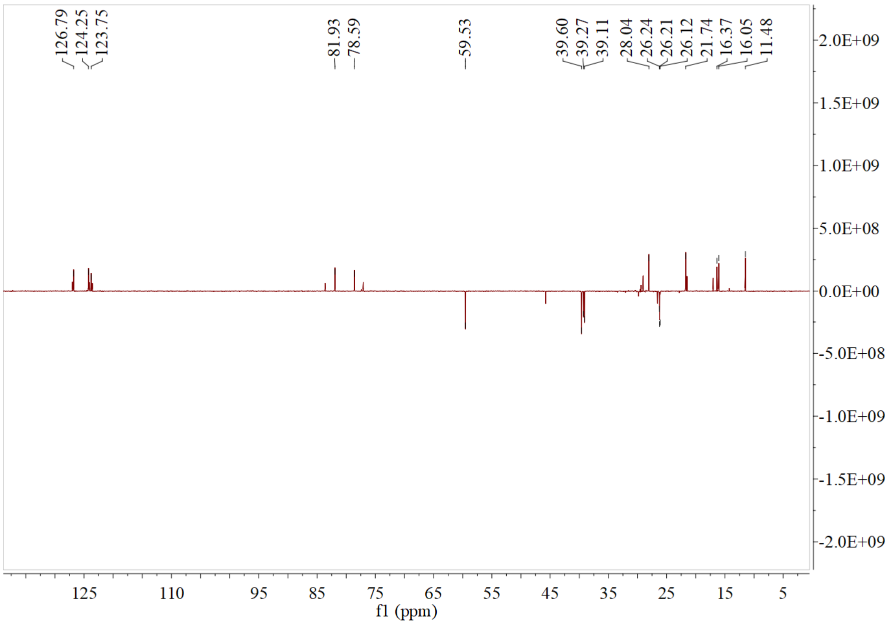

Figure S4. DEPT 135° spectrum of compound **1** in $CDCl_3$

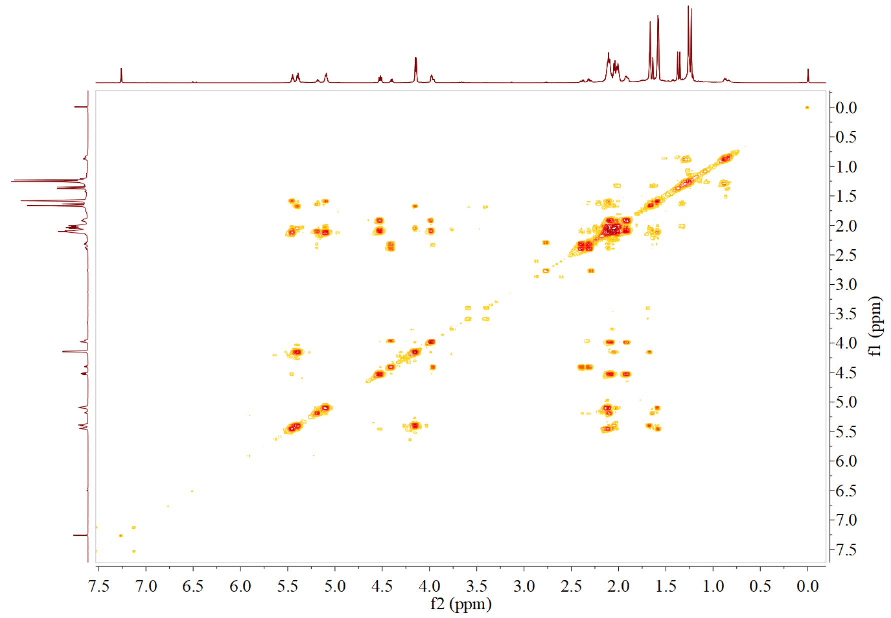

Figure S5. ^{1}H–^{1}H COSY spectrum of compound **1** in $CDCl_3$

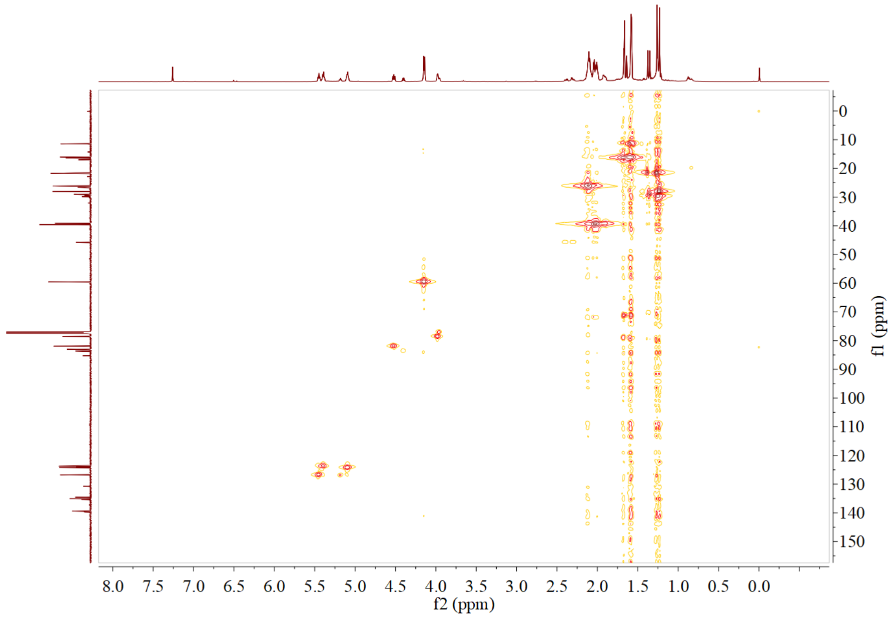

Figure S6. HMQC spectrum of compound **1** in $CDCl_3$

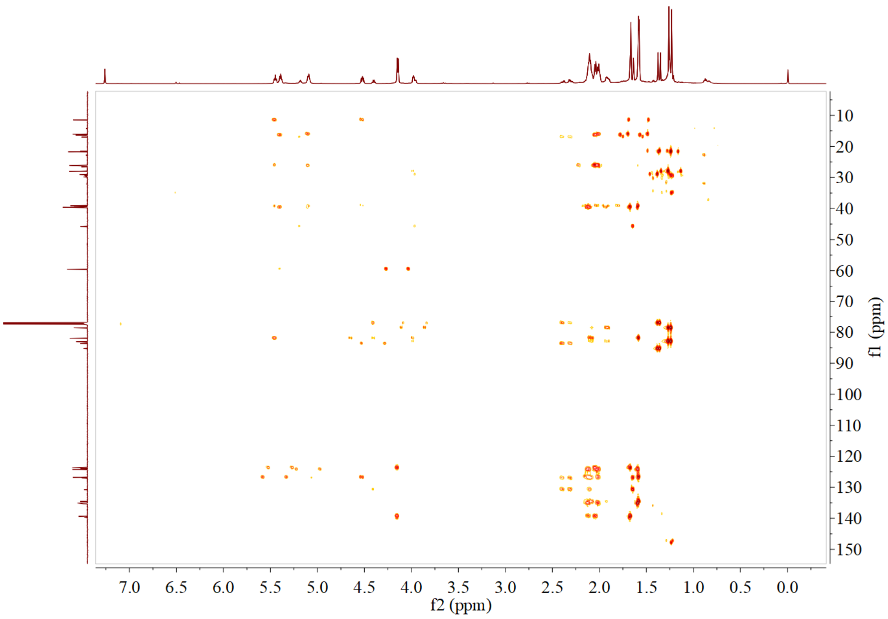

Figure S7. HMBC spectrum of compound **1** in $CDCl_3$

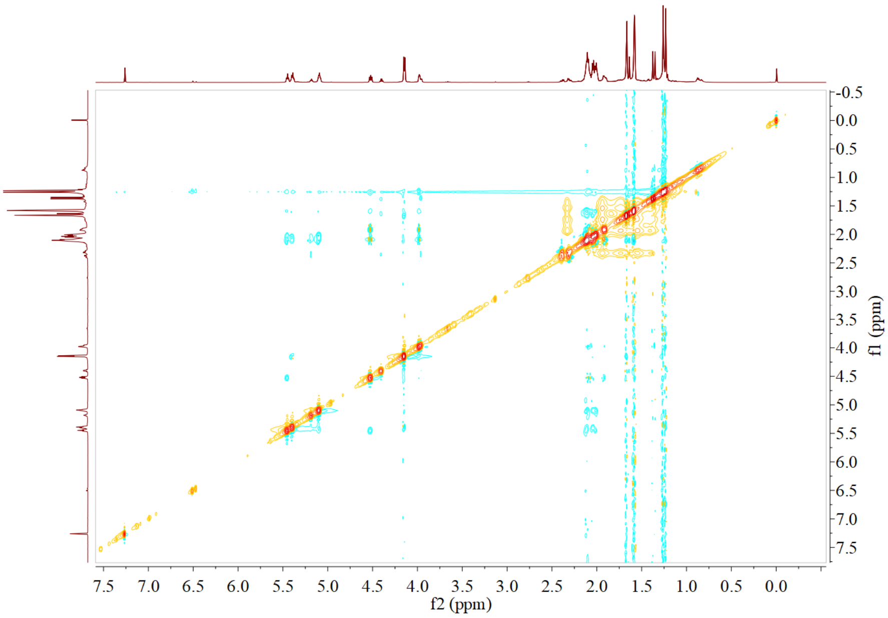

Figure S8. NOESY spectrum of compound **1** in $CDCl_3$

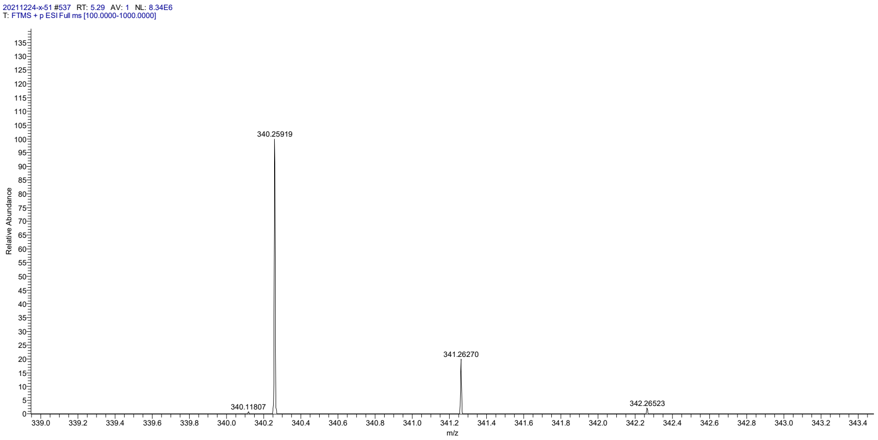

Figure S9. HR-ESI-MS of compound **1**

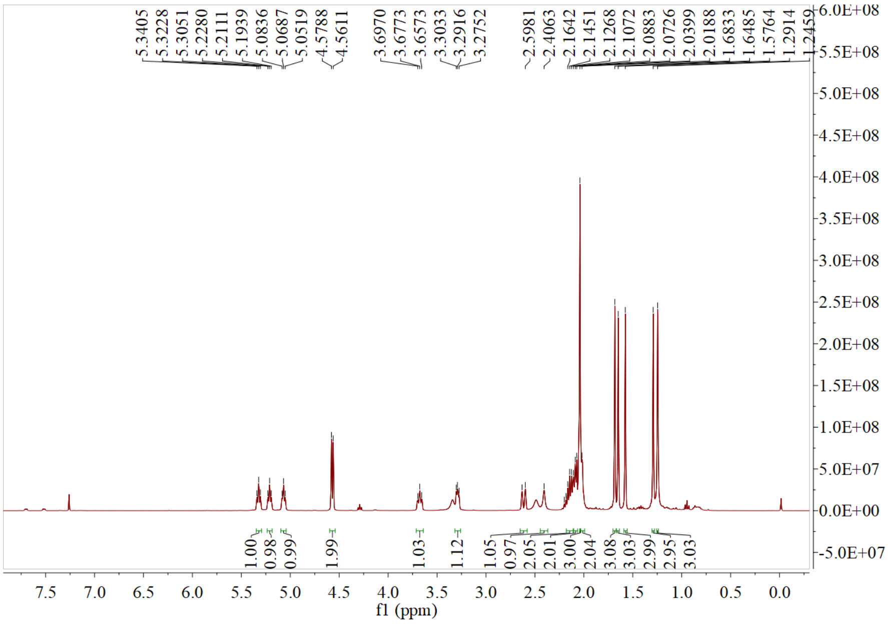

Figure S10. ^{1}H NMR spectrum of compound **2** in $CDCl_3$ (600 MHz)

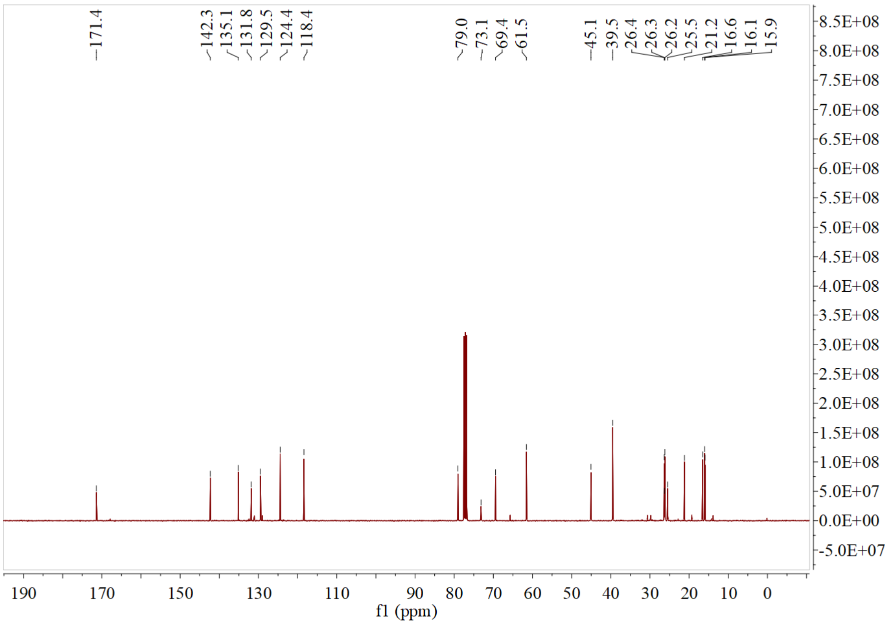

Figure S11. ^{13}C NMR spectrum of compound **2** in $CDCl_3$ (150 MHz)

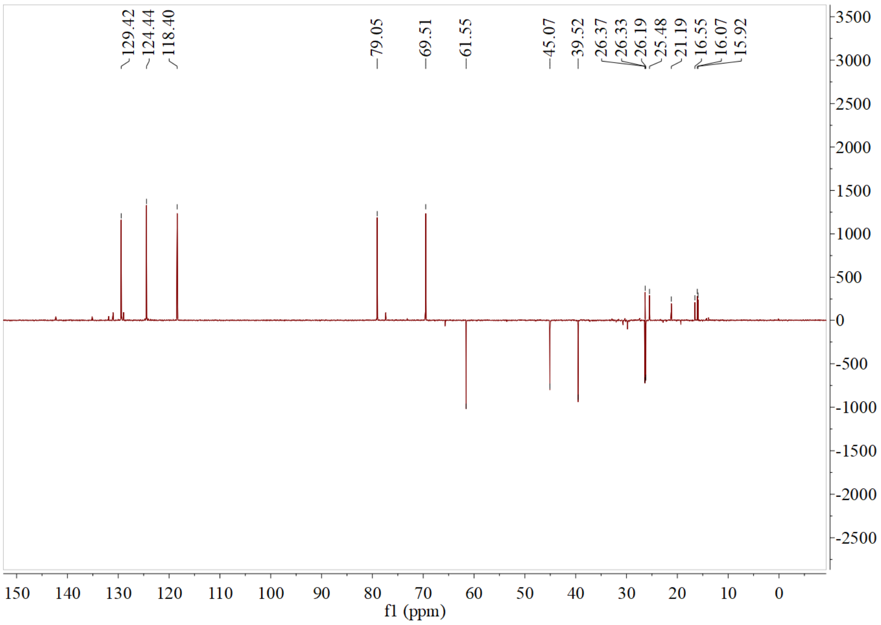

Figure S12. DEPT 135° spectrum of compound **2** in $CDCl_3$

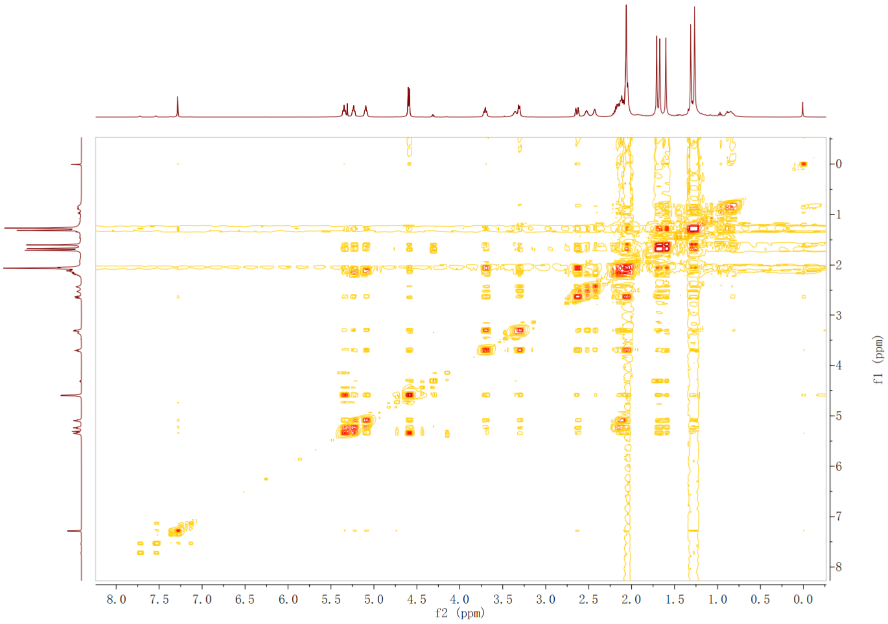

Figure S13. ^{1}H–^{1}H COSY spectrum of compound **2** in $CDCl_3$

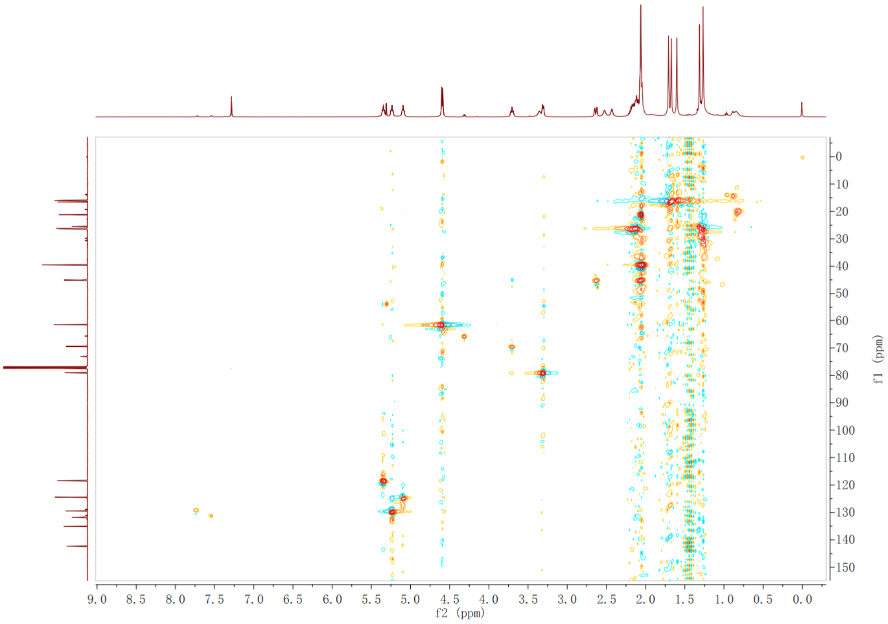

Figure S14. HMQC spectrum of compound **2** in $CDCl_3$

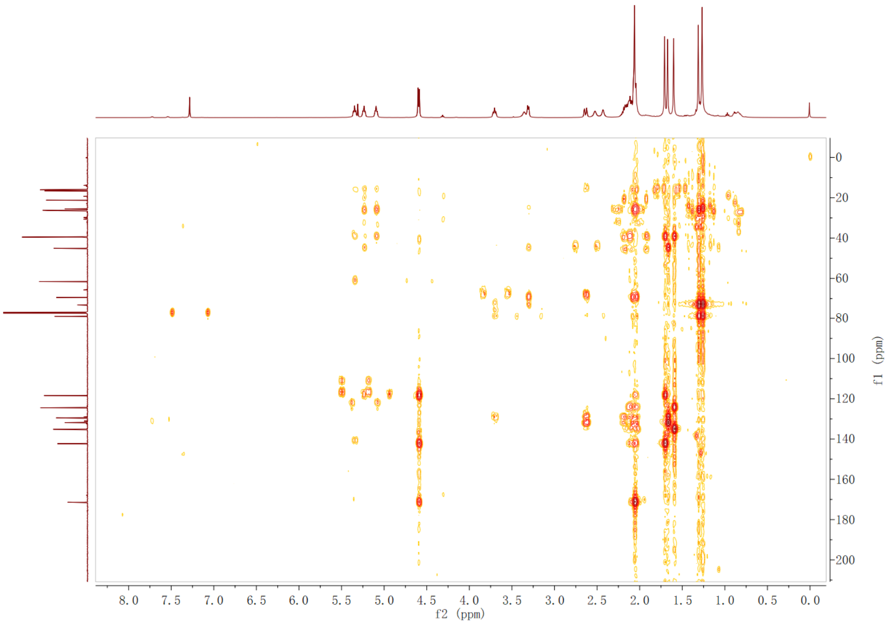

Figure S15. HMBC spectrum of compound **2** in $CDCl_3$

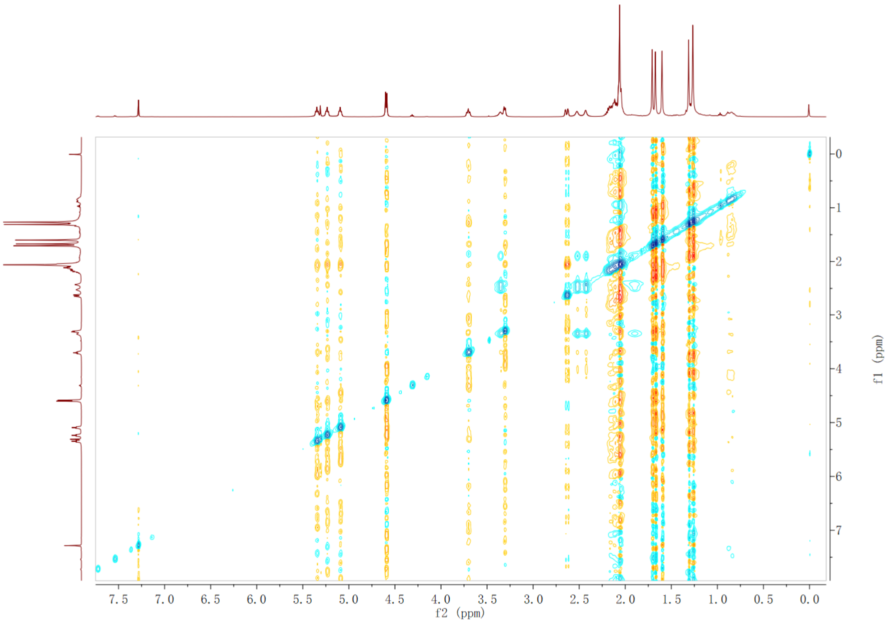

Figure S16. NOESY spectrum of compound **2** in $CDCl_3$

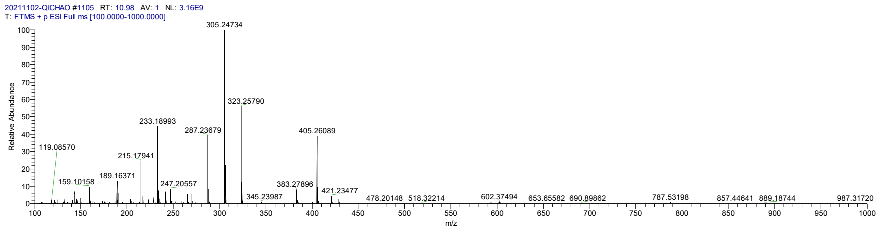

Figure S17. HR-ESI-MS of compound **2**

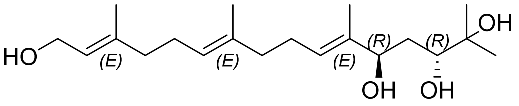

1a

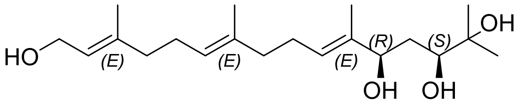

1b

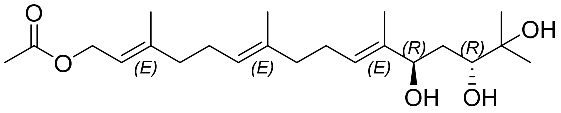

2a

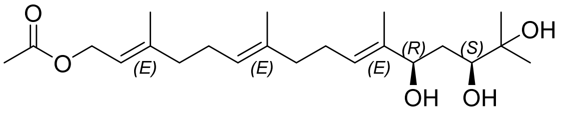

2b

Figure S18. Chemical structure of the calculated configurations.

Table S1. Statistics of ordinary least squares (OLS) linear regression of experimental and computed ^{13}C-NMR chemical shifts.

Configuration	DP4+ probability (%)	R^2	RMSE
**1a**	100	0.9924	4.28
**1b**	0	0.9905	4.63
**2a**	93.26	0.9951	3.73
**2b**	6.74	0.9958	3.53
